# Citizens, Research Ethics Committee Members and Researchers’ Attitude Toward Information and Consent for the Secondary Use of Health Data: Implications for Research Within Learning Health Systems

**DOI:** 10.1177/1556264621992214

**Published:** 2021-03-12

**Authors:** Annabelle Cumyn, Roxanne Dault, Adrien Barton, Anne-Marie Cloutier, Jean-François Ethier

**Affiliations:** 1 Groupe de Recherche Interdisciplinaire en Informatique de la Santé (GRIIS), Faculté de Médecine et des Sciences de la Santé, 7321 Université de Sherbrooke, Sherbrooke, Québec, Canada; 2 Département de Médecine, Faculté de Médecine et des Sciences de la Santé, 7321 Université de Sherbrooke, Sherbrooke, Québec, Canada; 3 Data Access Component, Quebec SPOR Support Unit, 98629 Université de Sherbrooke, Sherbrooke, Québec, Canada; 4 27051 Centre National de la Recherche Scientifique–Institut de Recherche en Informatique de Toulouse (CNRS–IRIT), Toulouse, Île-de-France, France

**Keywords:** health data, secondary use, learning health systems, survey, research ethics, informed consent

## Abstract

A survey was conducted to assess citizens, research ethics committee members, and researchers’ attitude toward information and consent for the secondary use of health data for research within learning health systems (LHSs). Results show that the reuse of health data for research to advance knowledge and improve care is valued by all parties; consent regarding health data reuse for research has fundamental importance particularly to citizens; and all respondents deemed important the existence of a secure website to support the information and consent processes. This survey was part of a larger project that aims at exploring public perspectives on alternate approaches to the current consent models for health data reuse to take into consideration the unique features of LHSs. The revised model will need to ensure that citizens are given the opportunity to be better informed about upcoming research and have their say, when possible, in the use of their data.

## Introduction

In the last decade, implementations of learning health systems (LHSs) are on the rise around the world (Lessard et al., 2017). LHSs focus on closely coupling clinical care with both the conduct of research and the translation of research into practice. Ensuing changes to care practice offer a new start for the next iteration of the cycle. Their development reflects the intention to use, on a large scale, people's health data generated on a daily basis within the healthcare system to facilitate the research and clinical continuum (Delaney et al., 2013; The Learning Healthcare Project, 2020). For example, LHSs aim to improve healthcare by allowing personalized models of care. They may also reduce the pressure on the healthcare system and its costs (Budrionis & Bellika, 2016). The endpoint is to fulfill the promise of evidence-based medicine by (1) enabling research pertinent to an organization or jurisdiction that rapidly generates new knowledge that can be swiftly applied in current clinical practice (changing the focus from research projects to learning cycle), (2) introducing a culture of shared responsibilities, and (3) facilitating the participation of citizens, patients, and healthcare providers in the production and dissemination of new knowledge (Budrionis & Bellika, 2016).

The province of Quebec (Canada) is currently working on the implementation of a learning health data platform named the “Learning Health and Social Services Research Platform” (*Plateforme apprenante pour la recherche en santé et services sociaux au Québec* in French, abbreviated “PARS3”) to support the secondary use of primary care health data in research as well as for other LHS activities, such as reflective practice and quality improvement (GRIIS, 2019). This platform will therefore enable (with proper authorizations, obviously) a more complete understanding of an individual, through her/his health data. While obvious sources include hospitals’ and clinics’ electronic health records, other important sources include research repositories or connected devices. This implies a shift of focus by putting the individual (rather than an organization) at the center of the system. This poses significant challenges as all these sources use heterogeneous technological systems. To solve this aspect, PARS3 relies on a clinical ontological model supporting semantic interoperability across various data systems (GRIIS, 2019). Access to this data will be highly regulated according to information security standards, and provision of the necessary access permissions such as research ethics committee (REC) approval for research.

In Canada, federal laws protect personal data and each province is responsible for the governance of healthcare. In the province of Quebec, access to hospital-based health data is regulated by the *Act respecting health services and social services*^1^ and consent to participation in research by the *Civil Code of Quebec.*^2^ Research in all Canadian institutions funded by three federal agencies must comply with the *Tri-Council Policy Statement: Ethical Conduct for Research Involving humans* (Government of Canada, 2019). Canadian and provincial normative and legal frameworks were, however, not established with learning platforms in mind. Several authors beyond the Canadian context have argued that current consent models are neither optimal for LHSs nor do they take into account the aspects inherent to LHSs like the requirement to go through knowledge transfer as part of the cycle, thereby increasing the likelihood of transformative impact on care (Faden et al., 2013b). The current standard of project-specific individual consent is inoperable on such a scale and would not enable us to achieve the social benefits of data reuse. Delegated consent for access to hospital-based health data, possible in specific contexts under the *Act respecting health services and social services*^1^, lacks provisions permitting healthcare users to be informed about the use of their health data and express their consent or dissent regarding their use for research or other purposes.

Thus, a major consideration in the implementation of an LHS is how to inform and support consent from individuals for the reuse of their health data for research with an approach that balances patient autonomy, transparency, and social benefits of data reuse. An exhaustive literature review performed in 2018 allowed us to analyze characteristics of different consent models that could be implemented within an LHS (Cumyn et al., 2019). It identified the meta-consent model proposed by Ploug and Holm (2016) as a very promising model. This model aims to be flexible by offering options for specific, broad, or blanket consent (or symmetric options for refusal), combined with dynamic consent (Ploug & Holm, 2016). The model has been proposed to answer the challenges of informed consent and registry-based or biobank research (Ploug & Holm, 2017).

With the start of the implementation of an LHS platform in Quebec, we were particularly interested to survey the opinions of concerned parties, and specifically citizens, regarding information and consent for the secondary use of health data for research. The objective of this study was to conduct a province-wide survey to examine Quebec citizens, REC members, and researchers’ points of view about the secondary use of health data for research, including the importance of information and consent as well as their attitude toward fundamental aspects of a new model of consent (meta-consent) adapted to LHSs. This survey was part of a mixed-method study to inform a series of focus groups to deepen our understanding of Quebec citizens’ opinion about the meta-consent model for the reuse of their health data for research.

## Method

### Sampling Strategy and Population

#### Citizens

A group of 50,000 phone numbers was randomly generated from a sampling frame owned by BIP Research polling firm, which contained 1,947,000 phone numbers and postal codes from the entire province of Quebec (BIP Research, 2015). This group consists of 75% landline numbers and 25% cell phone numbers. Using the six-digit postal code, the random group was then stratified by administrative region to represent the entire Quebec territory. Quotas were also used for gender, age, and education to ensure the inclusion of each of these key population subgroups in the sample. Only adults (≥18 years old) and residents of the province of Quebec were eligible to participate in the survey. For landline numbers, only one adult per household was asked to take part in the survey. As for cell phone numbers, the holder of the phone line was asked to participate if eligible.

From this group, the aim was to obtain 385 respondents. Our primary goal was to derive the characteristics of the general population. Classically, using a normal distribution of answers, a margin of error of 5%, a confidence interval of 95% (*z*-score of 1.96), and assuming a standard deviation of 0.5 for a population of 8.38 million (corresponding to the population of the province of Quebec in 2018), the suggested sample size is 385. Nevertheless, the questions are answered using a 5-point Likert scale and so these assumptions are unlikely to hold. This context has been evaluated before; a method has been proposed by Park and Jung (2009) and has been used in a context very similar to ours (Karbwang et al., 2018). This method suggests that assumptions from a traditional estimation sample size overestimate the sampling requirement in the context of answers given using a Likert scale and that a sample size of 231 would allow a margin of error of 5% given the estimated parameters. Even so, given a certain dose of uncertainty inherent to the estimation of parameters a priori, we decided to aim for the worst-case scenario and target 385 respondents.

#### Researchers and REC members

A convenience sampling method was used for researchers and REC members. Directors from 19 research centers and coordinators from 33 network REC members across the province of Quebec were contacted by our research team. Directors and coordinators, identified by a publicly available online list (Gouvernement du Québec, 2020a, 2020b), were asked to share an invitation to participate in an online version of the survey to their researchers and REC members. Overall, 8 research centers (42%) and 22 network RECs (67%) accepted to share the invitation with their teams. For the researchers, only those who have been primary investigators or co-investigators on a research project requiring the approval of a human REC were eligible to participate in the survey. The survey was programmed and self-administered online using the LimeSurvey software version 3.21.3. Multiple survey completions were avoided by the use of a unique URL for each survey. Data collection was anonymous.

### Survey Development and Content

The survey content was developed from themes previously identified in our scoping review (Cumyn et al., 2019). Although some prior research used scenario-based questionnaires, upon consultation with literacy experts, we chose a statement-based questionnaire that could be administered by phone and that would inform subsequent focus groups. Supported by an expert in methodology, the research team developed a 13-item survey using closed-ended statements. The full survey, as administered to the respondents, is presented in a supplemental file (Appendix 1). For the first 12 statements (S1-S12), respondents were asked to indicate their level of agreement on a 5-point Likert scale (“strongly agree,” “somewhat agree,” “neither agree nor disagree,” “somewhat disagree,” and “strongly disagree”). For the last statement (S13), respondents had to choose between two statements, whichever one they considered most important to them.

The 13-item survey covered five main domains investigating citizens, researchers, and REC members’ attitude toward:

- The secondary use of people's health data for research (S1). In this survey, the definition of health data provided to respondents was information included in electronic medical records such as any health diagnoses, test results, doctor and nurse notes, as well as drug prescriptions. Research with health data was defined as research that reuses already existing health information. We excluded clinical and prospective research from this definition.
- The level of information and consent acceptable regarding the use of de-identified health data and identifying health data in research (S2-S7). In this survey, identifying health data was defined as data that contains information that could permit direct identification (e.g., a postal address). This definition was chosen to reflect the occasional research project where health data needs to be coupled with other data such as environmental data.
  - *Scenario 1*. Information is provided regarding the use of people's health data and consent is requested.
  - *Scenario 2*. Information is provided regarding the use of people's health data, but consent is not requested.
  - *Scenario 3*. No information is provided regarding the use of people's health data and consent is not requested.
- The characteristics of a meta-consent model (S8-S10) are as follows:
  - the acceptability of the implementation of a secure website to allow citizens to be informed and to express their consent preferences for the secondary use of their health data for upcoming research projects;
  - the importance of support from health professionals for the use of the secure website; and
  - the acceptability of default settings that allow the use of health data according to the current laws when people did not express their consent preferences.
- The delegation of consent to a third party (S11-S12):
  - the delegation of consent to a director of professional services (current situation in Quebec for the secondary use of hospital health data for research); and
  - the delegation of consent to RECs (hypothetical situation).
- The relative valuation of individual control versus the societal benefits of research (S13).

### Survey Testing and Administration

#### Citizens

The survey was administered to Quebec citizens by BIP Research, a professional polling firm, between June 27, 2018 and July 7, 2018 (BIP Research, 2015). The survey was administered by phone by experienced interviewers previously trained for the purpose of this study. The interviews were conducted in both French and English. Prior to the official data collection, two recorded pretests were conducted with 10% of the sample to adjust the survey content and to standardize the administration between interviewers. These interviews were discarded.

#### Researchers and REC members

The web-based survey was administered to researchers and REC members between October 22, 2019 and December 20, 2019. During the data collection period, two reminders were sent to increase the response rate.

Two different methods of data collection were used, respectively, for citizens and researchers/REC members. For citizens, a phone administration was preferred over an online administration to promote the inclusion of people with a low literacy level (e.g., difficulties with reading). As for researchers and REC members, we opted for an online administration because it was the easiest way to reach these populations. Minor modifications were made between the phone and the web-based versions of the survey to adapt their respective formats. However, the content of the survey was identical between groups to avoid changing the meaning. Tests were conducted to ensure that a similar level of comprehension of survey content was obtained from the two administration methods.

### Statistical Analysis

Frequencies were used to describe the respondent characteristics and responses to each item of the survey. Citizen characteristics were compared with those of the entire Quebec population using chi-squared tests.

The difference in the point of view between the three groups (citizens, REC members, and researchers) was also assessed for each of the survey items. First, responses to each statement were pooled into three categories: agreement (“strongly agree” and “somewhat agree”), neutral (“neither agree nor disagree”), and disagreement (“strongly disagree” and “somewhat disagree”). Then, the difference in the level of agreement between the three groups was statistically compared using chi-squared tests or Fisher's exact tests. Two-tailed tests were used and *p*-values of ≤.05 were considered significant. Analyses were performed using SPSS IBM Statistics version 26.

## Results

### Citizens

The professional pooling firm used a random selection of 1,961 phone numbers and finally yielded a sample size of 387 Quebec adult citizen respondents. The response rate was 20% whereas the cooperation rate (rate of reached individuals who agreed to participate in the survey) was 38%. These percentages are similar to the ones observed in national U.S. random-digit dialed surveys (Fox, 2011; Kim et al., 2015). The interviews took an average of 14.3 min with a standard deviation of 2.66 min.

### Researchers and REC Members

For the web-based survey, 99 REC members and 66 researchers completed the questionnaire. The completion rate, namely the rate of respondents who completed questionnaires among those who attempted to complete it, was 75% (165 of 220 respondents). Among the 55 people who did not complete the survey, 29 are unidentified (they did not answer the first question in the questionnaire related to identification), 17 are researchers, and 9 are REC members.

#### Characteristics of the Respondents

Table 1 compares survey citizen respondents with the general Quebec population. Our respondents differed significantly from the 2018 statistics of the Quebec population as reported by the Institut de la Statistique du Québec (2018, 2019) in three aspects: age, level of education, and language. Our sample had a lower proportion of young people (18–44 years old: 28.2% vs. 41.7%) and a higher proportion of elderly (65 years old and more: 34.1% vs. 22.7%), which is often the case for surveys, as well as a lower proportion of individuals with no high school diploma (8.5% vs. 11.3%). In addition, there were more native French speakers in our sample compared with the entire population of Quebec (90.4% vs. 78.1%) even if the survey was administered in both French and English. All administrative regions of the Quebec province were represented in the survey, including both rural and urban areas (information not presented in Table 1). Most of the respondents never took part in a health research study (82.4%) and slightly more than half visited a hospital or a medical clinic three times or more in the last year for themselves or for a loved one.

**Table 1.** Characteristics of the Citizen Respondents Compared to the Population of the Province of Quebec (Canada).

	Respondents (*n* = 387) (%)	Quebec population^a^(%)	*p*
Gender			
Male	47.5	49.2	.515
Female	52.5	50.8	
Age			
18 to 44 years old	28.2	41.7	<.001
45 to 64 years old	37.7	35.5	
65 years old and more	34.1	22.7	
Higher diploma obtained			
No diploma	8.5	11.3	<.001
High school diploma or vocational studies diploma	36.5	34.8	
Collegial diploma	21.7	21.1	
University diploma	33.3	32.8	
Language spoken most often at home			
French only	90.4	78.1	<.001
English only	4.4	7.7	
French and English	2.8	0.8	
Other language	2.4	13.4	
Medical visits in the last 12 months for her/himself or a loved one		NA	NA
0	9.6		
1–2	39.3		
3 and more	51.1		
Actual or previous participation in a health research study		NA	NA
Yes	17.6		
No	82.4		

^a^ Statistics from the Institut de la statistique du Québec (2018, 2019).

Table 2 presents the characteristics of the REC members and the researchers. Thirty-eight percent of REC member respondents have a scientific role in their committee, 26% are public representatives, and 16% are ethicists. Health professionals, legal experts, coordinators as well as students were also represented in the sample. Close to 80% of them have two or more years of experience as active members of a REC. As for researchers, 83.3% have already used health data for their research projects as a principal investigator or a co-investigator.

**Table 2.** Characteristics of the REC Member and the Researcher Respondents.

	Total (*n* = 165) (%)	REC members (*n* = 99) (%)	Researchers (*n* = 66) (%)
Gender			
Male	39.4	38.4	40.9
Female	54.0	56.6	50.0
Did not want to answer	6.6	5.0	9.1
Age			
18 to 44 years old	38.2	39.4	36.3
45 to 64 years old	43.6	38.4	51.5
65 years old and more	13.3	18.2	6.1
Did not want to answer	4.9	4.0	6.1
Uses of health data for a research project	NA	NA	
Yes			83.3
No			16.7
Role in the REC	NA		NA
Scientist		38.4	
Citizen		26.3	
Ethicist		16.2	
Health professional		7.1	
Legal expert		5.0	
Coordinator		4.0	
Student		1.0	
Dual role		2.0	
Year of experience as a member of a REC	NA		NA
Less than 2 years		22.2	
2 years and more		77.8	

*Note*. REC = Research Ethics Committee.

#### What are the Respondents’ Opinions Regarding the Secondary Use of People's Health Data for Research?

**Figure 1.**
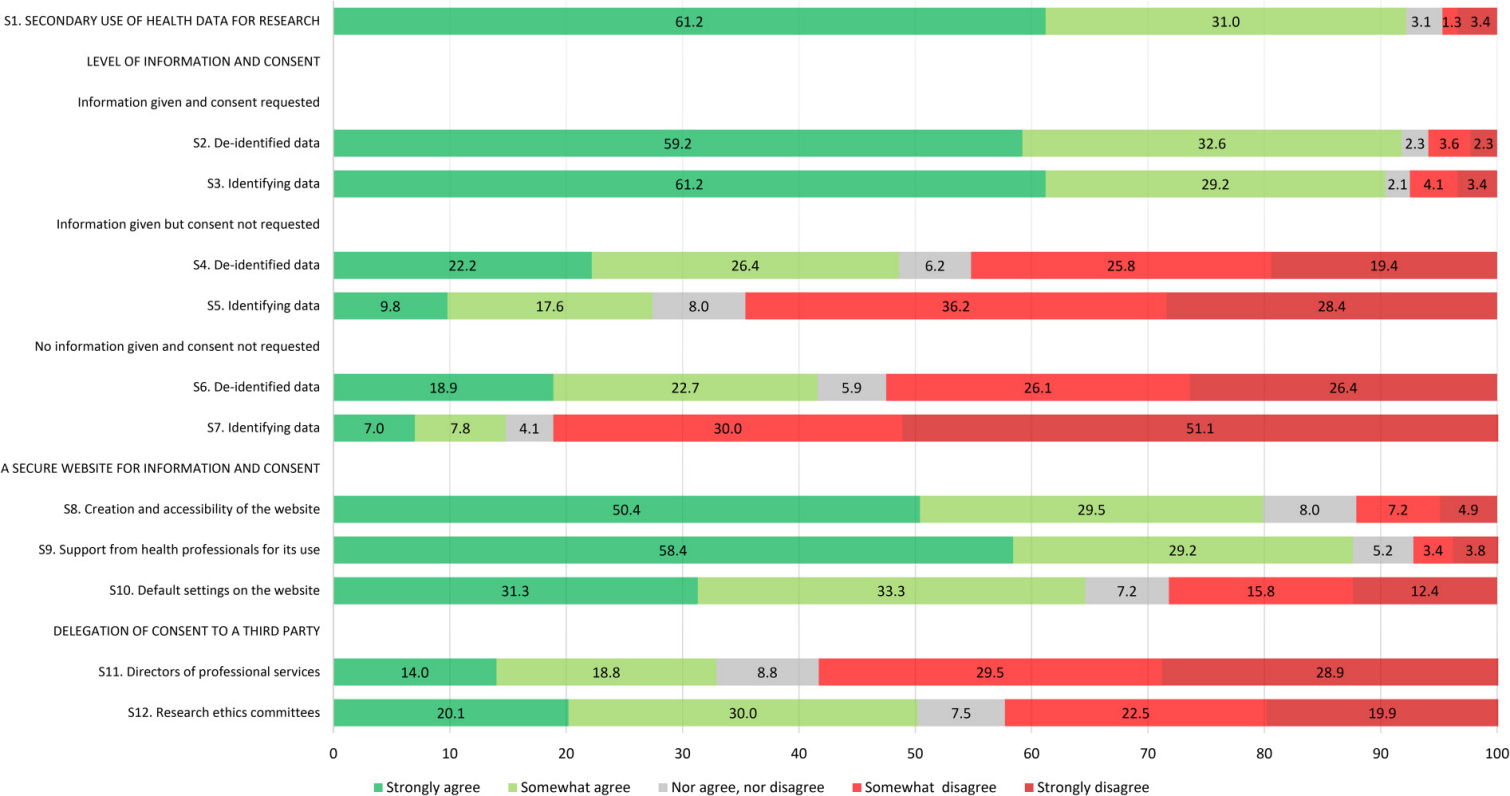
Level of agreement of citizen respondents toward each survey item.

**Figure 2.**
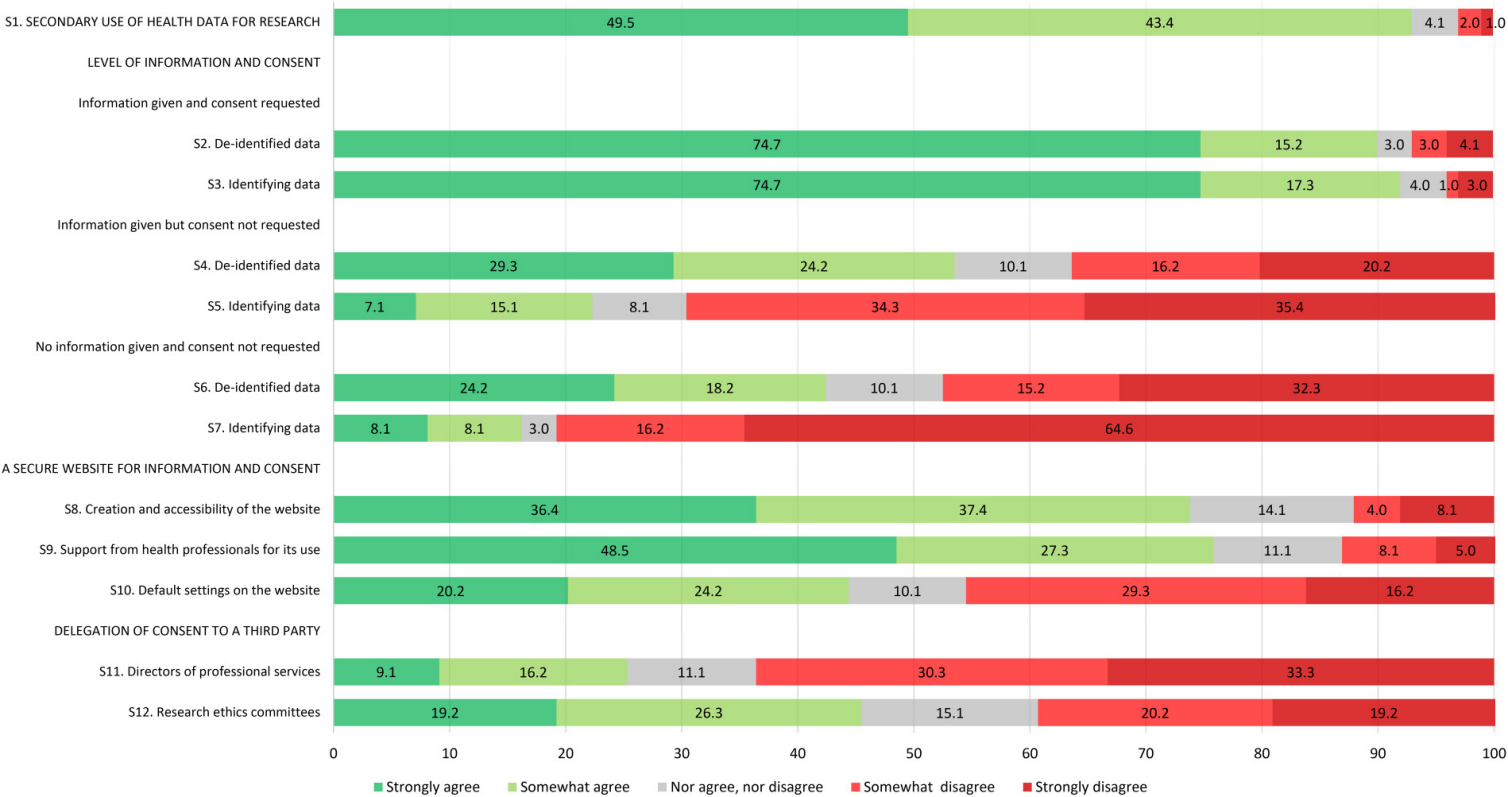
Level of agreement of REC member respondents toward each survey item.

**Figure 3.**
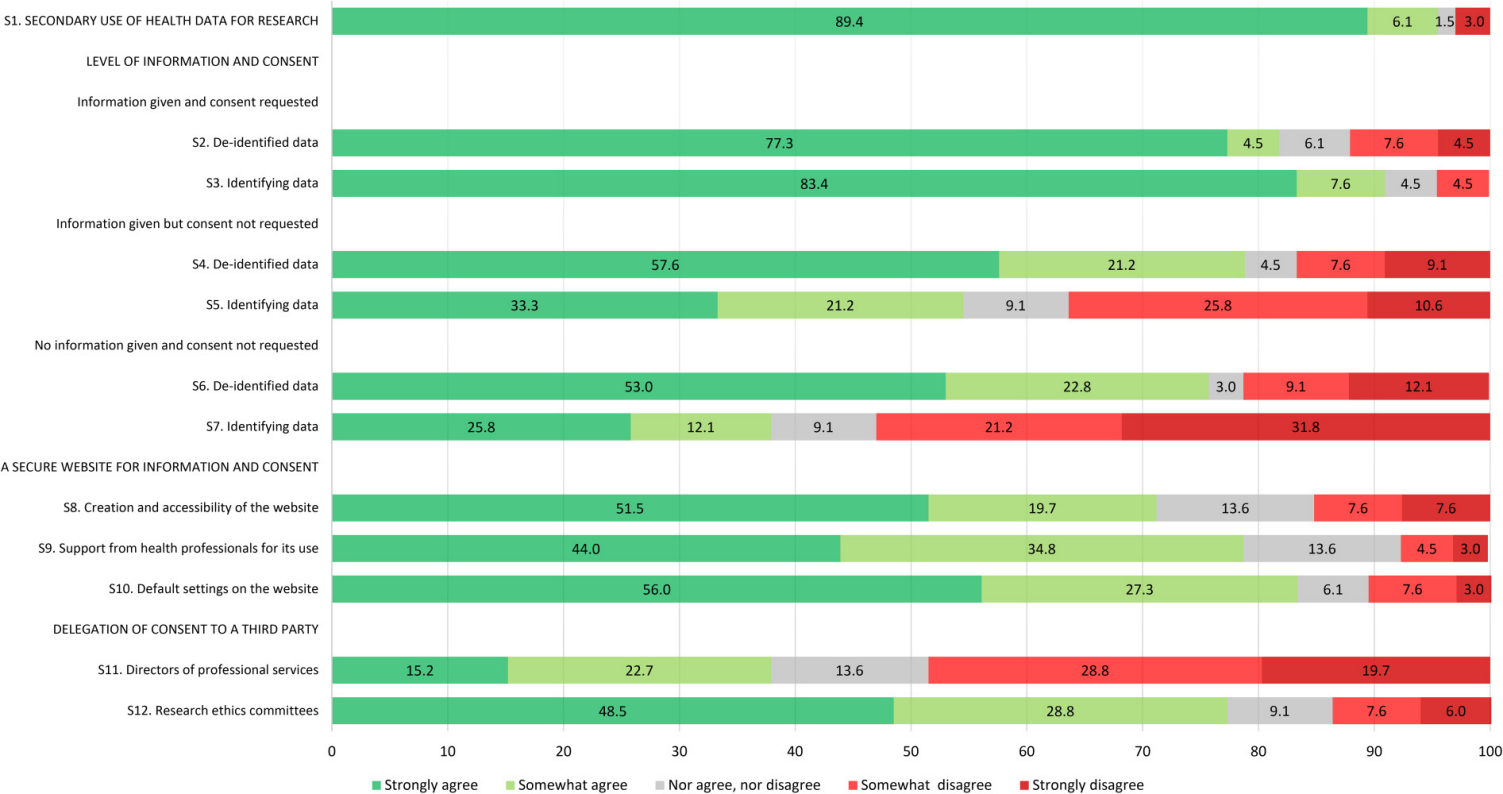
Level of agreement of researcher respondents toward each survey item.

Citizens were mostly supportive of the secondary use of people's health data for research to advance medical knowledge and improve patient care; 61.2% strongly agreed and 31.0% somewhat agreed with this statement while only a few respondents were neutral or disagreed (3.1% neither agreed nor disagreed, 1.3% somewhat disagreed, and 3.4% strongly disagreed) (Figure 1). REC members had a similar level of agreement but were more evenly split between strongly and somewhat agreed (49.5% strongly agreed and 43.4% somewhat agreed with the statement) (Figure 2) while researchers showed strong support (89.4% strongly agreed and 6.1% somewhat agreed with the statement) (Figure 3). Overall, all respondents surveyed were supportive of the general reuse of people's health data for research purposes to advance knowledge and improve care.

#### What are the Respondents’ Opinions Regarding the Level of Information and Consent for the Secondary Use of De-identified and Identifying Health Data for Research?

*Scenario 1. Information is provided and consent is requested:* Citizens considered acceptable to use people's health data for research, whether they are de-identified or identifying if people are informed about the use of their data and asked for their consent (respectively, 91.8% and 90.4% strongly or somewhat agreed with this statement) (Figure 1). REC members and researchers seemed to share a similar opinion to citizens, with, however, slightly higher proportions of respondents who are strongly in agreement with these statements (Figures 2 and 3). There was no statistical difference between the three groups of respondents for both de-identified and identifying data (*p* = .138 and .373, respectively) (Table 3).

**Table 3.** Comparison of the Citizen, REC Member, and Researcher Responses to the Survey.

	Citizens (*n* = 387)			REC members (*n* = 99)			Researchers (*n* = 66)			*p*
	Strongly/somewhat agree (%)	Neither agree nor disagree (%)	Strongly/somewhat disagree (%)	Strongly/somewhat agree (%)	Neither agree nor disagree (%)	Strongly/somewhat disagree (%)	Strongly/somewhat agree (%)	Neither agree nor disagree (%)	Strongly/somewhat disagree (%)	
*1. General attitude toward secondary use of health data for research*										
Secondary use of health data for research	92.2	3.1	4.7	92.9	4.1	3.0	95.5	1.5	3.0	.888
*2. Attitude toward the level of information and consent according to the type of data*										
*Information is provided and consent is requested*										
*De-identified data*	91.8	2.3	5.9	89.9	3.0	7.1	81.8	6.1	12.1	.138
*Identifying data*	90.4	2.1	7.5	92.0	4.0	4.0	91.0	4.5	4.5	.373
*Information is provided, but no consent is requested*										
*De-identified data*	48.6	6.2	45.2	53.5	10.1	36.4	78.8	4.5	16.7	<.001
*Identifying data*	27.4	8.0	64.6	22.2	8.1	69.7	54.5	9.1	36.4	<.001
*No information is provided, and no consent is requested*										
*De-identified data*	41.6	5.9	52.5	42.4	10.1	47.5	75.8	3.0	21.2	<.001
*Identifying data*	14.8	4.1	81.1	16.2	3.0	80.8	37.9	9.1	53.0	<.001
*3. Attitude toward a secure website to be informed regarding upcoming researches and to express consent preferences for the use of their health data*										
Creation and accessibility of the secure website	79.9	8.0	12.1	73.8	14.1	12.1	71.2	13.6	15.2	.250
Support from health professionals	87.6	5.2	7.2	75.8	11.1	13.1	78.8	13.6	7.6	.012
Default settings on the secure website	64.6	7.2	28.2	44.4	10.1	45.5	83.3	6.1	10.6	<.001
*4. Attitude toward delegation of consent to a third party*										
Current situation – DPS	32.8	8.8	58.4	25.3	11.1	63.6	37.9	13.6	48.5	.253
Hypothetical situation – REC	50.1	7.5	42.4	45.5	15.1	39.4	77.3	9.1	13.6	<.001

*Note*. DPS = Directors of Professional Services; REC = Research Ethics Committees.

*Scenario 2. Information is provided but consent is not requested:* For de-identified data, citizens gave polarized answers (22.2% strongly agreed, 26.4% somewhat agreed, 6.2% neither agreed nor disagreed, 25.8% somewhat disagreed, and 19.4% strongly disagreed) (Figure 1). However, when identifying data were concerned, citizens mainly disagreed with this statement (36.2% somewhat disagreed and 28.4% strongly disagreed). The opinion of REC members regarding de-identified data and identifying data in this context was very similar to citizens’ opinion (Figure 2). However, it appears that a larger proportion of researchers agreed (strongly or somewhat agreed) that people's de-identified or identifying health data could be used for research without their consent if they are informed about the use of their data (Figure 3). The differences in the proportions between the three groups of respondents were statistically significant: Researchers seemed more comfortable, compared to citizens and REC members, to use people's de-identified or identifying health data for research purposes when information is provided regarding data use, but consent is not sought (*p* < .001 for both de-identified and identifying data) (Table 3).

*Scenario 3. No information is provided and consent is not requested*: For this third scenario, the level of agreement of each group regarding de-identified health data was similar to the previous scenario (Figures 1, 2 and 3). In the case of identifying health data, acceptance across the three groups of respondents surveyed was low. It appears, however, that the difference in the level of agreement between the three groups is statistically significant: Again, researchers seemed more comfortable, compared to citizens and REC members, to use people's de-identified or identifying health data for research purposes without informing them and without seeking their consent (*p* < .001 for both de-identified and identifying data) (Table 3).

#### What are the Respondents’ Opinions Toward Certain Aspects of the Meta-consent Model?

*Creation and accessibility of a secure website to allow citizens to be informed and to express their consent preferences regarding the use of their health data for upcoming research projects*: Close to 80% of citizens showed a positive attitude toward the creation of such a secure website (50.4% strongly agreed and 29.5% somewhat agreed with this statement). Interestingly, REC members appeared to be less motivated than citizens towards such a website (36.4% strongly agreed and 37.4% somewhat agreed) and researchers showed a similar enthusiasm to that of citizens (51.5% strongly agreed and 19.7% somewhat agreed). However, there were no statistical differences between the three groups of respondents (*p* = .171).

*Support from health professionals for the use of the secure website*: A great majority of citizens (58.4% strongly agreed and 29.2% somewhat agreed) were in favor of health professionals providing support to patients for the use of the website, including help to express consent preferences. A great majority of REC members and researchers were also in agreement with this statement (Figures 2 and 3). However, it appeared to be significantly more important for citizens (87.6% of citizens strongly or somewhat agreed vs. 75.8% of REC members vs. 78.8% of researchers, *p* = .006) (Table 3).

*Acceptability of default settings on the secure website*: We asked the respondents if it was acceptable to use people's health data for research if they did not express their consent preferences regarding the use of their data, as long as Quebec's health data access regulations were satisfied. This statement was intended in part to test the acceptability of current regulations permitting retrospective research with record-based health data. In addition, a better understanding of citizen expectations would help to define default settings on the secure website. The presence of default settings corresponds to one of the six essential elements of a meta-consent model (Ploug & Holm, 2016). There was a strong divergence between the three groups of respondents regarding this statement. Only 64.6% of citizen respondents strongly or somewhat agreed with this statement. Only 44.4% of REC members agreed (20.2% strongly agreed and 24.2% somewhat agreed) with the existence of default settings other than “no” while on the contrary, a strong majority of researchers support it (56.0% strongly agreed and 27.3% somewhat agreed with this statement). The difference between the three groups was statistically significant (*p* < .001) (Table 3).

#### What are the Respondents’ Opinions Toward Delegation of Consent to a Third Party?

In the last set of statements, we presented two different scenarios in which we measured the level of agreement about delegation of consent to a third party for the use of people's health data for research. In the first statement, we evaluated the acceptability to delegate consent to a member of the hospital administration (director of professional services), which is allowed in Quebec for retrospective research with hospital health data, while in the second statement, we evaluated the acceptability to delegate consent to RECs. Only one-third of citizens were in agreement (14.0% strongly agreed and 18.8% somewhat agreed) with the delegation of consent to a director of professional services (i.e., with the current regulation). Half of the citizens agreed with the hypothetical scenario, which is to delegate consent to RECs (20.1% strongly agreed and 30.0% somewhat agreed) (Figure 1). It appears that the current situation in the province of Quebec whereby a member of the hospital administration may grant authorization to access hospital-based health data for research is similarly not well accepted by the other respondents (Figures 2 and 3). There was no general agreement from REC members regarding REC's hypothetical decisional power to decide, on people's behalf, the use of their health data for research purposes (19.2% strongly agreed, 26.3% somewhat agreed, 15.1% neither agreed nor disagreed, 20.2% somewhat disagreed, and 19.2% strongly disagreed) (Figure 2). On the other hand, a majority of researchers would support this hypothetical scenario (48.5% strongly agreed and 28.8% somewhat agreed with this statement) (Figure 3). The difference observed between the three groups of respondents was statistically significant (*p* < .001) (Table 3).

#### Which Value, Between Individual Control on Personal Data and Common Good Through Medical Research, is the Most Important for the Respondents?

For the last part of the survey, we asked the respondents to choose between two statements that intended to evaluate which value was the most important for them between individual control on their data (which is related to autonomy) and fostering common good through easier medical research. Citizens expressed more agreement with the statement relating to individual control (61.5%) than the one relating to the common good (37.5%), suggesting that they value their control more than the facilitation of research (Table 4). Like citizens, the majority of REC members expressed more agreement with the statement relating to individual control (63.6%). On the contrary, researchers expressed more agreement with the statement relating to the common good (69.7%).

**Table 4.** Respondents’ Attitude Regarding Individual Control and Common Good.

*Question:* Which statement is more important to you?	*Individual control:* It is more important that each citizen may easily decide whether his or her health data is used for research	*Common good:* It is more important that the citizens’ health data be easily accessible for research	*Refuse to take a position*
Citizens (*n* = 387)	238 (61.5%)	145 (37.5%)	4 (1.0%)
REC members (*n* = 99)	63 (63.6%)	25 (25.3%)	11 (11.1%)
Researchers (*n* = 66)	13 (19.7%)	46 (69.7%)	7 (10.6%)

*Note*. REC = Research Ethics Committee.

## Discussion

This survey had two main objectives: (1) Draw a current portrait of the attitude of Quebec citizens, REC members, and researchers regarding the reuse of people's health data for research as well as regarding certain components of a new consent model (meta-consent) better fitting an LHS; (2) inform and guide a series of focus groups to further explore components of the meta-consent model for its eventual implementation in the Quebec LHS platform. Several interesting observations can be made on our results.

First, Quebec citizens strongly support the reuse of their health data for research to enhance knowledge and improve care, which is aligned with similar surveys conducted in another Canadian province (Willison et al., 2007) and in the United States (Cho et al., 2015; Kelley et al., 2015; Kim et al., 2015; Kraybill et al., 2016). However, citizens do not seem to have a positive opinion regarding waiving consent. In fact, the vast majority of them supported the reuse of health data (de-identified and identifying) in research when two conditions are met: (1) People are informed about the use of their data and (2) consent is requested. Less than half supported the use of health data (de-identified and identifying) if people are informed about the use of their data, but no consent is requested. As presented in our scoping review (Cumyn et al., 2019), the literature on consent in LHSs emphasizes the central role of the information and consent processes in maintaining trust for the use of data for research. In fact, several studies showed that even if there is good acceptance from the public for research with health data, there is a strong desire for transparency and to be asked for permission before using this information. As illustrated in our scoping review, participants expressed a preference for an “ask me each time” model of consent in research (Flory et al., 2016; Kaplan et al., 2016; Kass et al., 2016; Kelley et al., 2015; Nayak et al., 2015; Sugarman & Califf, 2014; Weinfurt et al., 2016; Whicher et al., 2015). However, Cho et al. (2015) noted that when the consent process impacts significantly the feasibility of the research, participants were willing to accept less elaborate approaches. The literature seemed to suggest that when the trade-offs are better understood by participants, what matters most to the public may be being informed directly and retaining some degree of decision-making control (for instance an initial broad consent) (Cho et al., 2015; Willison et al., 2007), which is compatible with a meta-consent model, rather than a more intrusive and cumbersome study-specific consent process (Simon et al., 2011; Thiel et al., 2014; Weinfurt et al., 2016).

It is interesting to note that REC members had opinions more aligned with citizens than with researchers in this survey regarding the use of health data for research without informing and obtaining consent from people for both de-identified and identifying data. This finding is important, as one of the roles of RECs is the protection of research participants. An alignment with citizens’ views might be relevant to achieve this objective. Researchers seem to support more strongly the use of de-identified health data without informing people nor having their consent. As the use of de-identified health data is crucial for certain research projects for which consent cannot always be obtained, this result is not very surprising. Several researchers and ethicists described specific individual consent with opt-in as impractical, as it would be too time-consuming and expensive to ask consent from every patient for every research project (Faden et al., 2013a; Hoffman & Podgurski, 2012; Kaplan et al., 2016). A few seem to believe that this approach to consent would fit well the needs of LHSs. Nevertheless, the researchers substantially agreed with a delegated consent model to RECs which might indicate a fair level of trust in RECs by researchers despite having different opinions on some issues.

More citizen respondents agreed with the use of de-identified data than with the use of identifying data if the information is not provided and people's consent is not sought (41.6% strongly or somewhat agreed vs. 14.8%). Similar observations were made in several other studies (Kaplan et al., 2016; Kim et al., 2015; Willison et al., 2007). In their qualitative study, Mayo et al. (2017) showed that participants accept that researchers and hospitals (for most uses) would access their data but would be nervous at the idea that drug companies or insurance companies would have access to “even some of those data.” Patients may fear their data could be used for wrongful reasons. For example, they may fear that insurance companies could have access to medical data and deny coverage on this basis. This concern could decrease people's consent to the general use of their data, especially their identifying data, for all research purposes. We posit that a lack of information may lead to a magnification of such concerns (Cumyn et al., 2019).

The vast majority of our citizen respondents found it important that a website be created to allow citizens to be notified of the use of their health data for upcoming research projects and to express their consent preferences on this matter. It appears that Quebec citizens would like to have control over their health data and seem to be open to use a web-based platform to manage its use. This finding is aligned with the fundamental principle of the meta-consent model, which is a form of dynamic consent based on a computer-based technology (Ploug & Holm, 2016). As shown in several studies, dynamic consent appears to promote participation in research by informed and scientifically literate participants (Daniel & Choquet, 2016) as well as increase participants’ trust (Staa et al., 2016; Williams et al., 2015). Many studies considered it to be practical, respectful, and supportive of patient autonomy (Angrist & Jamal, 2015; Shelton, 2011; Williams et al., 2015).

The citizen respondents strongly agreed with the importance of healthcare professionals supporting people in their use of the website and helping them to better understand the different consent choices proposed on the website. These results confirm that the findings in our scoping review (Cumyn et al., 2019) seem applicable to the province of Quebec. Indeed, many studies highlighted the important role of the healthcare provider, especially the physicians, in the information and consent processes regarding participation in research activities. In a study by Kelley et al. (2015), participants strongly prefer a notification or consent process led by their physician rather than by researchers or other clinical staff. In a similar study (Cho et al., 2015), 84.5% of patients surveyed preferred to be asked permission to participate in the medical records review study by their physician as opposed to by a researcher or research nurse not involved in their care. However, the participation of healthcare professionals in a consent process may come at a cost. Further research will be needed to study issues related to practicability, engagement from healthcare professionals, and alignment with ethical principles and regulations.

A majority of citizen respondents agreed with the use of people's health data according to default settings that are aligned with current privacy laws. Default settings are one of the six essential elements of a meta-consent model (Ploug & Holm, 2016). Default settings allow individual control while having the potential to promote research, as individuals can change their settings at any time according to their preferences. As a system of default settings is difficult to understand, especially in a survey with little information, it will be important to further explore its understanding in the upcoming focus groups. It seems that no other studies have evaluated attitudes toward this characteristic of meta-consent.

Citizen respondents generally expressed low acceptability regarding the fact that a third party (e.g., a member of the hospital management) could decide on the people's behalf the use of their health data for research purposes. In the same vein, interestingly, REC members and researchers did not wholeheartedly agree either with the delegation of consent to a hospital administrator. This result shows that the public, as well as the other concerned parties, seem to disagree with the models of delegated consent that are applied in some contexts, including in Quebec for retrospective studies with hospital health data. Citizens seem to want to retain control over their health data. The creation of a secure website to allow citizens to have control over their data would build trust: People will be more willing to share their data if they know when they are used, for what purposes, and what the impact of this research on improving care is.

## Limitations

Our findings should be interpreted with some caveats in mind. First of all, we are aware of the potential selection bias related to the use of the BIP Research sampling frame to generate our study sample and the possible impact of this bias on the generalizing of our findings to the entire province of Quebec. Also, we are aware that telephone surveys have their own challenges such as nonresponse bias, particularly that response rates have been on a precipitous decline (Curtin et al., 2005). However, our results still provide a good starting point for analyzing the opinions of Quebec citizens on issues surrounding consent for the use of health data in research and are part of a larger sequential mixed-methods study. Also, the impact of data access is likely to be stronger for older citizens given the higher probability that they receive health care from a health organization, and so that health data is generated.

Secondly, the use of closed-ended questions limits the interpretation of the results, since it was not possible for respondents to qualify their answers. Because of that, our results should be interpreted with some caution considering that they provide only a partial picture of the attitudes of the various concerned parties. For example, with regard to the default settings, it was difficult to present the possible nuances associated with them. Some respondents might have considered that these settings would automatically permit the use of data when, in fact, a default setting could be an “ask me each time” setting. For that reason, further research following a qualitative methodology will be necessary to explore this perception in depth.

Thirdly, contextualization within the LHS framework was not done explicitly in the survey because this concept is complex and unknown to a majority of study participants. It is possible that answers to statements could have varied if specific contexts had been proposed. Nevertheless, it was important to have such a survey to characterize the current situation and opinions of the participants to inform future changes. The survey is part of a larger sequential mixed-methods study and will be used to inform the content of focus groups in which the LHS context will be made explicit.

Fourthly, we asked for respondents’ opinions regarding research in general. Their attitudes might have been different if we had presented them with more specific research scenarios (e.g., academic research vs. private research). Since this is more difficult to assess in a survey, we will explore these concepts in the focus groups.

Finally, we are aware that our survey did not capture all aspects related to the meta-consent model and does not provide a comprehensive picture of the opinions of citizens, researchers, and REC members on this topic.

### Best Practices

Based on our findings, Quebec citizens largely support the use of their health data for research to advance knowledge and improve care, but they value transparency and they place high importance on personal control on the use of their health data for research. In that regard, our results suggest that they would support the development of a web-based platform to be informed about incoming research projects and to express their consent preferences regarding the use of their health data.

For these reasons, policymakers need to consider the public opinion regarding the use of their health data for research. In addition, they need to take into consideration the evolution of our healthcare system into an LHS. Accordingly, changes to current consent and data access models will need to be carefully considered.

### Research Agenda

This survey is part of a larger project that explores public perspectives on alternate approaches to the current consent models in Quebec to take into consideration the unique features of an LHS. This survey as well as the focus groups will lead to key recommendations that will support and guide decision-makers at local and provincial levels in future decisions on this issue. The ultimate goal is to review and adjust the model of consent in Quebec, where needed. This model will need to ensure that citizens have the opportunity to be better informed about incoming researches with their health data and have their say, when possible, in the use of their information.

### Educational Implications

A dialogue must be initiated with front-line professionals to explore their perception about being actively involved in the process of informing their patients about research with health data and in supporting their decision-making regarding consent for the use of their health data for research. We need to define their roles and responsibilities in the possible deployment of meta-consent in Quebec.

## Supplemental Material

sj-docx-1-jre-10.1177_1556264621992214 – Supplemental material for Citizens, Research Ethics Committee Members and Researchers’ Attitude Toward Information and Consent for the Secondary Use of Health Data: Implications for Research Within Learning Health Systems Click here for additional data file. Supplemental material, sj-pdf-1-eeg-10.1177_1556264621992214 for Citizens, Research Ethics Committee Members and Researchers’ Attitude Toward Information and Consent for the Secondary Use of Health Data: Implications for Research Within Learning Health Systems by Annabelle Cumyn, Roxanne Dault, Adrien Barton, Anne-Marie Cloutier and Jean-François Ethier in Journal of Empirical Research on Human Research Ethics

## Notes

1. *Act respecting health services and social services*, CQLR, c S-4.2.

2. *Civil Code of Quebec*, CQLR, c CCQ-1991, s 20.

## References

[bibr1-1556264621992214] Angrist M. Jamal L. (2015). Living laboratory: Whole-genome sequencing as a learning healthcare enterprise. Clinical Genetics, 87(4), 311–318. 10.1111/cge.12461 25045831 PMC4302048

[bibr2-1556264621992214] BIP Research (2015). BIP research . https://www.bipresearch.com/

[bibr3-1556264621992214] Budrionis A. Bellika J. G. (2016). The learning healthcare system: Where are we now? A systematic review. Journal of Biomedical Informatics, 64, 87–92. 10.1016/j.jbi.2016.09.018 27693565

[bibr4-1556264621992214] Cho M. K. Magnus D. Constantine M. Lee S. S.-J. Kelley M. Alessi S. Korngiebel D. James C. Kuwana E. Gallagher T. H. Diekema D. Capron A. M. Joffe S. Wilfond B. S. (2015). Attitudes toward risk and informed consent for research on medical practices: A cross-sectional survey. Annals of Internal Medicine, 162(10), 690–696. 10.7326/M15-0166 25868119 PMC4776759

[bibr5-1556264621992214] Cumyn A. Barton A. Dault R. Cloutier A.-M. Jalbert R. Ethier J.-F. (2019). Informed consent within a learning health system: A scoping review. Learning Health Systems, 4(2), e10206. 10.1002/lrh2.10206 32313834 PMC7156861

[bibr6-1556264621992214] Curtin R. Presser S. Singer E. (2005). Changes in telephone survey nonresponse over the past quarter century. Public Opinion Quarterly, 69(1), 87–98. 10.1093/poq/nfi002

[bibr7-1556264621992214] Daniel C. Choquet R. (2016). Clinical research informatics contributions from 2015. Yearbook of Medical Informatics, 1, 219–223. 10.15265/IY-2016-044 PMC5171544 27830254

[bibr8-1556264621992214] Delaney B. C. Ethier J.-F. Curcin V. Corrigan D. Friedman C. (2013). *International perspectives on the digital infrastructure for The Learning Healthcare System*, 2013. Washington, DC: AMIA conference. 10.13140/2.1.3015.6165

[bibr9-1556264621992214] Faden R. Kass N. Whicher D. Stewart W. Tunis S. (2013a). Ethics and informed consent for comparative effectiveness research with prospective electronic clinical data. Medical Care, 51(8 Suppl. 3), S53–S57. 10.1097/MLR.0b013e31829b1e4b 23793051

[bibr10-1556264621992214] Faden R. R. Kass N. E. Goodman S. N. Pronovost P. Tunis S. Beauchamp T. L. (2013b). An ethics framework for a learning health care system: A departure from traditional research ethics and clinical ethics. Hastings Center Report, 43(s1), S16–S27. 10.1002/hast.134 23315888

[bibr11-1556264621992214] Flory J. H. Mushlin A. I. Goodman Z. I. (2016). Proposals to conduct randomized controlled trials without informed consent: A narrative review. Journal of General Internal Medicine, 31(12), 1511–1518. 10.1007/s11606-016-3780-5 27384536 PMC5130947

[bibr12-1556264621992214] Fox S. (2011). The social life of health information, 2011. 45.

[bibr13-1556264621992214] Gouvernement du Québec (2020a). Centres et réseaux—Fonds Santé . http://www.frqs.gouv.qc.ca/la-recherche/la-recherche-financee-par-le-frqs/centres-et-reseaux?field=all&grouptype=1&submit=Rechercher

[bibr14-1556264621992214] Gouvernement du Québec (2020b). Répertoire des ressources du réseau de la santé et des services sociaux en éthique et autorisation des recherches—Aperçu de l’éthique en santé et services sociaux—Professionnels de la santé—MSSS . https://www.msss.gouv.qc.ca/professionnels/ethique/ethique-en-sante-et-services-sociaux/repertoires/

[bibr15-1556264621992214] Government of Canada (2019, April 1). Tri-Council Policy Statement: Ethical Conduct for Research Involving Humans—TCPS 2 (2018) . https://ethics.gc.ca/eng/policy-politique_tcps2-eptc2_2018.html

[bibr17-1556264621992214] GRIIS (2019). PARS3 . GRIIS. https://griis.ca/en/solutions/pars3/

[bibr18-1556264621992214] Hoffman S. Podgurski A. (2012). Balancing Privacy, Autonomy, and Scientific Needs in Electronic Health Records Research (SSRN Scholarly Paper ID 1923187). *Social Science Research Network*. https://papers.ssrn.com/abstract=1923187

[bibr19-1556264621992214] Institut de la statistique du Québec (2018). Le bilan démographique du Québec. Édition 2018 . 174.

[bibr20-1556264621992214] Institut de la Statistique du Québec (2019). Répartition de la population de 25 à 64 ans selon le plus haut niveau de scolarité atteint, la région administrative, l’âge et le sexe, Québec et région administratives, 1990 à 2018 . http://www.stat.gouv.qc.ca/statistiques/education/niveau-scolarite/repartition-scol-ra-sexe-age.html#tri_tertr=50040000000000000&tri_sexe=1&tri_age=365&tri_stat=8403

[bibr21-1556264621992214] Kaplan S. H. Gombosev A. Fireman S. Sabin J. Heim L. Shimelman L. Kaganov R. Osann K. E. Tjoa T. Huang S. S. (2016). The patient’s perspective on the need for informed consent for minimal risk studies: Development of a survey-based measure. AJOB Empirical Bioethics, 7(2), 116–124. 10.1080/23294515.2016.1161672

[bibr22-1556264621992214] Karbwang J. Koonrungsesomboon N. Torres C. E. Jimenez E. B. Kaur G. Mathur R. Sholikhah E. N. Wanigatunge C. Wong C.-S. Yimtae K. Abdul Malek M. Ahamad Fouzi L. Ali A. Chan B. Z. Chandratilake M. Chiew S. C. Chin M. Y. C. Gamage M. Gitek I ., … FERCAP Multi-Country Research Team (2018). What information and the extent of information research participants need in informed consent forms: A multi-country survey. BMC Medical Ethics, 19(1), 79. 10.1186/s12910-018-0318-x 30219106 PMC6139128

[bibr23-1556264621992214] Kass N. Faden R. Fabi R. E. Morain S. Hallez K. Whicher D. Tunis S. Moloney R. Messner D. Pitcavage J. (2016). Alternative consent models for comparative effectiveness studies: Views of patients from two institutions. AJOB Empirical Bioethics, 7(2), 92–105. 10.1080/23294515.2016.1156188

[bibr24-1556264621992214] Kelley M. James C. Alessi Kraft S. Korngiebel D. Wijangco I. Rosenthal E. Joffe S. Cho M. K. Wilfond B. Lee S. S.-J. (2015). Patient perspectives on the learning health system: The importance of trust and shared decision making. American Journal of Bioethics, 15(9), 4–17. 10.1080/15265161.2015.1062163 PMC4821628 26305741

[bibr25-1556264621992214] Kim K. K. Joseph J. G. Ohno-Machado L. (2015). Comparison of consumers’ views on electronic data sharing for healthcare and research. Journal of the American Medical Informatics Association, 22(4), 821. 10.1093/jamia/ocv014 25829461 PMC5009901

[bibr26-1556264621992214] Kraybill A. Dember L. M. Joffe S. Karlawish J. Ellenberg S. S. Madden V. Halpern S. D. (2016). Patient and physician views about protocolized dialysis treatment in randomized trials and clinical care. AJOB Empirical Bioethics, 7(2), 106–115. 10.1080/23294515.2015.1111272 27833931 PMC5098473

[bibr27-1556264621992214] The Learning Healthcare Project (2020). Learning health care . http://www.learninghealthcareproject.org/section/background/learning-healthcare-system

[bibr28-1556264621992214] Lessard L. Michalowski W. Fung-Kee-Fung M. Jones L. Grudniewicz A. (2017). Architectural frameworks: Defining the structures for implementing learning health systems. Implementation Science, 12(1), 78. 10.1186/s13012-017-0607-7 28645319 PMC5481948

[bibr29-1556264621992214] Mayo R. M. Summey J. F. Williams J. E. Spence R. A. Kim S. Jagsi R. (2017). Qualitative study of oncologists’ views on the CancerLinQ rapid learning system. Journal of Oncology Practice, 13(3), e176–e184. 10.1200/JOP.2016.016816 28118106

[bibr30-1556264621992214] Nayak R. K. Wendler D. Miller F. G. Kim S. Y. H. (2015). Pragmatic randomized trials without standard informed consent?: A national survey. Annals of Internal Medicine, 163(5), 356–364. 10.7326/M15-0817 26215125 PMC5573142

[bibr31-1556264621992214] Park J. W. Jung M. S. (2009). A note on determination of sample size for a Likert scale. Communications for Statistical Applications and Methods, 16(4), 669–673. 10.5351/CKSS.2009.16.4.669

[bibr32-1556264621992214] Ploug T. Holm S. (2016). Meta consent—a flexible solution to the problem of secondary use of health data. Bioethics, 30(9), 721–732. 10.1111/bioe.12286 27628305 PMC5108479

[bibr33-1556264621992214] Ploug T. Holm S. (2017). Eliciting meta consent for future secondary research use of health data using a smartphone application - a proof of concept study in the Danish population. BMC Medical Ethics, 18(1), 51. 10.1186/s12910-017-0209-6 28810914 PMC5558710

[bibr34-1556264621992214] Shelton R. H. (2011). Electronic consent channels: Preserving patient privacy without handcuffing researchers. Science Translational Medicine, 3(69), 69cm4. 10.1126/scitranslmed.3002037 21307300

[bibr35-1556264621992214] Simon C. M. L’Heureux J. Murray J. C. Winokur P. Weiner G. Newbury E. Shinkunas L. Zimmerman B. (2011). Active choice but not too active: Public perspectives on biobank consent models. Genetics in Medicine, 13(9), 821–831. 10.1097/GIM.0b013e31821d2f88 21555942 PMC3658114

[bibr36-1556264621992214] van Staa T. P. Goldacre B. Buchan I. Smeeth L. (2016). Big health data: The need to earn public trust. BMJ (Clinical research ed.), 354, i3636. 10.1136/bmj.i3636 27418128

[bibr37-1556264621992214] Sugarman J. Califf R. M. (2014). Ethics and regulatory complexities for pragmatic clinical trials. JAMA, 311(23), 2381–2382. 10.1001/jama.2014.4164 24810723

[bibr38-1556264621992214] Thiel D. B. Platt T. Platt J. King S. B. Kardia S. L. R. (2014). Community perspectives on public health biobanking: An analysis of community meetings on the Michigan BioTrust for Health. Journal of Community Genetics, 5(2), 125–138. 10.1007/s12687-013-0162-0 23893769 PMC3955459

[bibr39-1556264621992214] Weinfurt K. P. Bollinger J. M. Brelsford K. M. Crayton T. J. Topazian R. J. Kass N. E. Beskow L. M. Sugarman J. (2016). Patients’ views concerning research on medical practices: Implications for consent. AJOB Empirical Bioethics, 7(2), 76–91. 10.1080/23294515.2015.1117536 27800531 PMC5085261

[bibr40-1556264621992214] Whicher D. Kass N. Faden R. (2015). Stakeholders’ views of alternatives to prospective informed consent for minimal-risk pragmatic comparative effectiveness trials. Journal of Law, Medicine & Ethics, 43(2), 397–409. 10.1111/jlme.12256 26242962

[bibr41-1556264621992214] Williams H. Spencer K. Sanders C. Lund D. Whitley E. A. Kaye J. Dixon W. G. (2015). Dynamic consent: A possible solution to improve patient confidence and trust in how electronic patient records are used in medical research. JMIR Medical Informatics, 3(1), e3. 10.2196/medinform.3525 25586934 PMC4319083

[bibr42-1556264621992214] Willison D. J. Schwartz L. Abelson J. Charles C. Swinton M. Northrup D. Thabane L. (2007). Alternatives to project-specific consent for access to personal information for health research: What is the opinion of the Canadian Public? Journal of the American Medical Informatics Association, 14(6), 706–712. 10.1197/jamia.M2457 17712084 PMC2213476

