# Supplementary material for: Citizens, Research Ethics Committee Members and Researchers’ Attitude Toward Information and Consent for the Secondary Use of Health Data: Implications for Research Within Learning Health Systems
Source: J Empir Res Hum Res Ethics. 2021 Mar 12;16(3):165–78. doi: 10.1177/1556264621992214 (PMC8236664; doi:10.1177/1556264621992214)
Supplement: sj-docx-1-jre-10.1177_1556264621992214 – Supplemental material for Citizens, Research Ethics Committee Members and Researchers’ Attitude Toward Information and Consent for the Secondary Use of Health Data: Implications for Research Within Learning Health Systems [file sj-docx-1-jre-10.1177_1556264621992214.docx]

**Supplementary File**

**Appendix 1. Survey as administered online on the LimeSurvey platform**

**CONTEXT**

Currently in Quebec, there is little research based on health data because of difficulties in accessing this data.

The health data we are referring to is the information included in citizens’ health records such as health diagnoses, test results and prescribed medication.

Increased use of this health data for research would advance knowledge and improve patient care.

Here, when we talk about research, we are talking about research projects that use existing health data. We are not talking about clinical research that evaluates new drugs or treatments.

For example, the use of health data could allow to determine, for a woman with a diagnosis of breast cancer, the most appropriate type of treatment according to her personal characteristics.

The purpose of this survey is to know your opinion about how citizens’ health data can be used for research.

**INTRUCTIONS**

This survey contains 13 statements.

For each of these statements, you will have to indicate if you “strongly agree”, “somewhat agree”, “neither agree nor disagree”, “somewhat disagree” or “strongly disagree”.

**SURVEY**

**Statement 1.** It is acceptable to use citizens’ health data for research to advance knowledge and improve care.

| Strongly agree | Somewhat agree | Neither agree nor disagree | Somewhat disagree | Strongly disagree |
| --- | --- | --- | --- | --- |

______________________________________________________________________________

To answer research questions, researchers sometimes have to use some personal information such as the date of birth or the postal code.

For example, it is necessary to use the postal code to identify the location of citizens at risk of heart problems during extreme cold spells to provide better and adapted care for them.

Whereas for other research questions, it is not necessary to use the citizens’ personal information. For instance, when we simply want to know the percentage of the Quebec population that received the influenza vaccine this year.

For the next three statements, imagine that a research project is currently underway. To answer the research question of this project, the researchers **need to** use some personal information.

Knowing this:

**Statement 2.** It is acceptable to use the citizens’ health data, which **include** certain personal information, if:

- the citizens **are** informed about the research project **and**
- they are **asked** for their permission to use their data.

| Strongly agree | Somewhat agree | Neither agree nor disagree | Somewhat disagree | Strongly disagree |
| --- | --- | --- | --- | --- |

**Statement 3.** It is acceptable to use the citizens’ health data, which **include** certain personal information, if:

- the citizens **are** informed about the research project **but**
- they are **not asked** for their permission to use their data.

| Strongly agree | Somewhat agree | Neither agree nor disagree | Somewhat disagree | Strongly disagree |
| --- | --- | --- | --- | --- |

**Statement 4.** It is acceptable to use the citizens’ health data, which **include** certain personal information, if:

- the citizens **are not** informed about the research project **and**
- they are **not asked** for their permission to use their data either.

| Strongly agree | Somewhat agree | Neither agree nor disagree | Somewhat disagree | Strongly disagree |
| --- | --- | --- | --- | --- |

______________________________________________________________________________

You will now read the same three statements, but this time, imagine that the researchers **do not need** to use personal information to answer their research question.

Knowing this:

**Statement 5.** It is acceptable to use the citizens’ health data, which **do not include** personal information, if:

- the citizens **are** informed about the research project **and**
- they are **asked** for their permission to use their data.

| Strongly agree | Somewhat agree | Neither agree nor disagree | Somewhat disagree | Strongly disagree |
| --- | --- | --- | --- | --- |

**Statement 6.** It is acceptable to use the citizens’ health data, which **do not include** personal information, if:

- the citizens **are** informed about the research project **but**
- they are **not asked** for their permission to use their data.

| Strongly agree | Somewhat agree | Neither agree nor disagree | Somewhat disagree | Strongly disagree |
| --- | --- | --- | --- | --- |

**Statement 7.** It is acceptable to use the citizens’ health data, which **do not include** personal information, if:

- the citizens **are not** informed about the research project **and**
- they are **not asked** for their permission to use their data either.

| Strongly agree | Somewhat agree | Neither agree nor disagree | Somewhat disagree | Strongly disagree |
| --- | --- | --- | --- | --- |

______________________________________________________________________________

For the next three statements, we will ask your opinion about the creation of a secure website.

Imagine that through this secure website, each citizen will be able to decide for herself or himself:

- If she or he wants to be informed about upcoming research projects based on health data **and;**
- If she or he agrees that her or his health data is used for certain research projects.

Note that this website would only intend to make personal choices. Under no circumstances, the citizens’ health data would appear on this website.

Knowing this:

**Statement 8.** It is important that such a secure website be created and accessible.

| Strongly agree | Somewhat agree | Neither agree nor disagree | Somewhat disagree | Strongly disagree |
| --- | --- | --- | --- | --- |

**Statement 9.** It is important that citizens can discuss with their healthcare provider to better understand the choices proposed on the secure website.

| Strongly agree | Somewhat agree | Neither agree nor disagree | Somewhat disagree | Strongly disagree |
| --- | --- | --- | --- | --- |

**Statement 10.** If a citizen has not made her or his choices on the secure website, it is acceptable to use her or his health data for research in accordance with the Quebec’s data protection laws.

| Strongly agree | Somewhat agree | Neither agree nor disagree | Somewhat disagree | Strongly disagree |
| --- | --- | --- | --- | --- |

______________________________________________________________________________

For the next two statements, we will ask your opinion on who should decide the use of health data for research.

**Statement 11.** It is acceptable for a member of the hospital management to decide on the citizens’ behalf if their health data will be used for research.

| Strongly agree | Somewhat agree | Neither agree nor disagree | Somewhat disagree | Strongly disagree |
| --- | --- | --- | --- | --- |

**Statement 12.** It is acceptable for an ethics committee to decide on the citizens’ behalf if their health data will be used for research.

| Strongly agree | Somewhat agree | Neither agree nor disagree | Somewhat disagree | Strongly disagree |
| --- | --- | --- | --- | --- |

**Statement 13.** Please indicate which one of this two statement is more important to you:

| It is more important that each citizen may easily decide whether his or her health data is used for research. | It is more important that the citizens’ health data be easily accessible for research. | Refuse to take position. |
| --- | --- | --- |
